# Local Anæsthesia in General Medicine and Surgery

**Published:** 1886-12

**Authors:** 


### BOOK REVIEWS.

LOCAL ANESTHESIA IN GENERAL MEDICINE AND SURGERY, *Being the Practical Application of the Author's Discoveries. By* J. LEONARD CORNING, M.D., *pp. 103. New York:* D. APPLETON & COMPANY. *Chicago :* A. C. MCCLURG & COMPANY. *1886.*

Nearly everything of value or of interest in this book had first appeared in the medical periodicals of the United States. It is principally a compilation of articles written by other men. It is entertaining and instructive, but the way in which the author forces his own personality upon the reader at every page is inexcusable. The book is one of the most flagrant examples which has appeared, of the growing tendency among certain of the members of the medical profession to attempt to advance their personal interests by exhibitions of egotisms, as is shown throughout this book by the frequent use of the nominative case of the first person singular, and the devising and displaying of needless modifications of instruments.

It has been ascertained that in using cocaine for producing local anæsthesia the following precautions are desirable : 1. The particular sample of the drug used must be a good one. The author prefers that prepared by Mariani, but others seem to be equally good. 2. Strong solutions, particularly about the head, are apt to produce unpleasant physiological symptoms. The author prefers a one or two per-centum solution. In one case local insensibility was maintained for one hour and a half by the use of a one-third per-centum solution, heated to 99° F. before injection. Heating the solution has been found to increase its anæsthetic power. 3. The best method in using the drug is, to first apply an Esmarch bandage, say in an extremity, up to the lower margin of the region which it is desired to render anæsthetic. Then using a solution of the desired strength, make repeated injections of say two minims, just under the skin, at short intervals of space throughout the area to be anæsthetized. Next apply the bandage above but not over the injected area, and apply the tourniquet. The bandage may or may not be removed below the point of operation. When the operation involves the deeper tissues, the injections of one to three drops each, are to be repeated at successively deeper points as the anæsthesia proceeds, even down to and under the periosteum. 4. The anæsthesia seems to continue as long as the circulation in the part is stopped. A practical suggestion in this connection is, that when large quantities of the drug have been used, the circulation in the part should be re-established gradually. One convenient method for this purpose is to leave an elastic band so adjusted as to retard only the subcutaneous venous circulation.

5. In addition to the use of cocaine in cutting operations, it has been found to be of great service for purposes of diagnosis and reduction of fractures and dislocations. In this class of cases the injections must be carried down to the bone, and the use of the tourniquet is desirable. In the cases reported all pain was abolished and no injurious effects resulted. 6. It is desirable to avoid the puncture of large veins. For this purpose the author uses superficial pressure enough to retard the venous circulation, and then traces the course of the veins with a soft blue pencil.

